# ASO Author Reflections: All Positive, Not All Necessary: Reconsidering Axillary Lymph Node Dissection When Every Sentinel Node is Positive

**DOI:** 10.1245/s10434-025-18275-9

**Published:** 2025-09-04

**Authors:** Walker Lyons, Austin D. Williams

**Affiliations:** Department of Surgical Oncology, Fox Chase Cancer Center, Philadelphia, PA USA

## Past

The management of axillary lymph nodes (LNs) in breast cancer treatment has undergone a significant evolution in the past 30 years. While axillary lymph node dissection (ALND) was historically the standard of care for any patient with breast cancer, there has been a trend towards less axillary surgery to decrease the morbidity that comes with ALND. The advent of sentinel lymphadenectomy (SLNB) in the 1990s reduced morbidity, particularly lymphedema, by sparing patients unnecessary dissection when nodes were negative.^1^ The landmark American College of Surgeons Oncology Group Z0011 (Z0011) and European After Mapping of the Axilla: Radiotherapy Or Surgery (AMAROS) trials extended this principle, showing that patients with one or two positive sentinel lymph nodes (SLNs) could safely avoid ALND without compromising survival or local control.^2,3^ Nonetheless, there is hesitation to omit ALND in patients with fewer than three SLNs retrieved and all are positive due to the concern that additional axillary disease may be left behind.

## Present

This question is one that continues to provoke discussion at institutional tumor boards, and on larger scales such as on the American Society of Breast Surgeons online forum. To address it directly, we analyzed the National Cancer Database for female patients with cT1–3N0 breast cancer who underwent SLNB between 2018 and 2021, grouping them by the number of positive/removed SLNs.^4^ ALND was performed in 11% of patients overall but was performed in 41% of patients with 2/2 positive SLNs and 26% of patients with 1/1 positive SLNs. Additional positive LNs were found on ALND in 56% and 40% of these groups, respectively. On multivariate analysis, having 2/2 positive SLNs was the strongest predictor of ALND. Adjuvant treatment patterns revealed another layer: despite similar distributions of 21-gene Recurrence Scores (Oncotype DX, Genomic Health), there were increased rates of adjuvant chemotherapy for hormone receptor (HR)-positive, human epidermal growth factor receptor 2-negative (HER2−) patients who were older than 50 years of age among patients who underwent ALND compared with those who did not undergo ALND. With a median follow-up of 35.4 months, ALND was not associated with improved overall survival (OS) in any SLN group.

## Future

Our findings highlight persistent variation in practice, with ALND still being performed at higher-than-expected rates in patients with 1–2 positive SLNs despite multiple randomized trials showing it does not improve survival or recurrence. Decisions to proceed with ALND in patients with 1/1 or 2/2 positive SLNs should involve multidisciplinary discussion, weighing how the discovery of additional positive nodes might influence systemic therapy. While our prior work has shown ALND should not be used solely to determine eligibility for CDK4/6 inhibitors, the specific impact on other treatment decisions should be carefully considered for each patient.^5^ In this study, we were unable to evaluate whether ALND changed treatment or OS in the clinically relevant group of HR+/HER2− patients older than 50 years of age with a Recurrence Score ≤25, a clinically relevant subgroup, due to the small sample size.

Future studies should clarify whether ALND meaningfully alters outcomes for patients in whom fewer than three SLNs are retrieved and all are positive by incorporating disease-free and recurrence-specific endpoints. Until then, the safety of de-escalation continues to be supported in this patient population: all positive does not mean all necessary, especially when the evidence tells us less can be more.
